# Latent profile analysis and influencing factors of female sexual function in IVF-ET in china

**DOI:** 10.1371/journal.pone.0356758

**Published:** 2026-08-26

**Authors:** Zhengyao Che, Qian Yang, Lirong Xia, Xinhui Wang, Wenhui Yang, Yu Ma, Huaimei Bi

**Affiliations:** 1 926th Hospital of Joint Logistics Support Force of PLA, Yunnan, China; 2 Yunnan Provincial Hospital of Traditional Chinese Medicine, Yunnan, China; 3 Yunnan College of Business Management, Yunnan, China; Qingdao University, CHINA

## Abstract

**Objective:**

To investigate the current status of female sexual function in IVF-ET, analyze the potential differences in sexual function characteristics among different categories of IVF-ET women, and analyze their associated factors, in order to provide reference for the development of hypothesis-driven sexual dysfunction screening strategies.

**Method:**

From January to April 2024, a sexual function survey was conducted on 305 female patients who underwent IVF-ET assisted pregnancy at a tertiary hospital in Yunnan Province, China. A self-made general information questionnaire, female sexual function index, and the 10-item Kessler Psychological Distress Scale were used, and latent profile analysis was conducted on the patient's sexual function.

**Result:**

The sexual function of 305 female IVF-ET patients can be divided into three categories: low sexual function group (21.0%), moderate sexual function group (40.9%), and good sexual function group (38.1%). IVF-ET women with younger age, higher education level, and psychological distress are more likely to be included in the moderate sexual function group; The probability of IVF-ET women residing in cities belonging to the group with good sexual function is high; The shorter the duration of infertility and the fewer pregnancies, the less likely it is to be included in the low sexual function group.

**Conclusion:**

There is heterogeneity in the sexual function of IVF-ET women, and these findings may help medical staff identify at-risk populations based on the associated factors of sexual function in different categories of patients, though prospective validation is needed.

## 1 Introduction

The global incidence of infertility is between 10% and 15%, and it is on the rise. The situation of infertility in China is even more severe, with an infertility rate of up to 25% among couples of childbearing age [1,2]. In vitro fertilization embryo transfer (IVF-ET) has become an effective measure for treating infertility. Due to uncertain treatment outcomes and traditional beliefs, female patients undergoing IVF-ET may experience adverse physical and mental symptoms such as sexual dysfunction and anxiety [3].

Sexual activity, also known as sexual life, refers to the sexual contact such as hugs, kisses, caresses, and intercourse that is carried out to meet the physical and mental sexual needs of mature individuals [4]. It is the first step toward achieving spontaneous conception and a key part of sexual quality and health. However, for infertile patients, it is difficult for them to enjoy or carry out this process normally, and their concern about whether they will eventually become pregnant weakens their experience in behavior, increasing the risk of sexual dysfunction [5]. Female sexual dysfunction (FSD) refers to a type of disease in which one or several stages of the female sexual response cycle are disrupted, affecting normal sexual activity. It mainly includes decreased libido, difficulty in sexual arousal, difficulty in achieving orgasm, poor vaginal wetting, low sexual satisfaction, and painful intercourse [6]. FSD poses a serious threat to sexual health and reduces the quality of sexual life. Studies have shown that sexual dysfunction is one of the causes of infertility, and a series of psychological problems caused by infertility can exacerbate sexual dysfunction and affect the treatment of infertility [7]. Due to the influence of traditional Chinese beliefs and cultural backgrounds, healthcare professionals and IVF-ET patients may be hesitant to discuss sexual history or sexual concerns. Therefore, there is a lack of research on female sexual function related to IVF-ET, and the evaluation criteria are mostly based on scale scores, ignoring individual differences, which may lead to a lack of targeted intervention plans in the later stages.

Latent profile analysis (LPA) is an individual centered approach that classifies individuals based on their response patterns on a scale, and has unique advantages in determining their category and differences [8]. Unlike traditional variable-centered approaches that assume homogeneous populations, LPA allows for the identification of heterogeneous subgroups with distinct sexual function profiles, which is particularly valuable for understanding the diversity of sexual function among IVF-ET patients and developing personalized care strategies. Therefore, this study is based on latent profile analysis, taking female patients undergoing IVF-ET as the research object to explore whether there is heterogeneity in their sexual function. Based on this, the associated factors of potential categories of sexual function are analyzed, in order to provide a basis for developing hypothesis-driven screening approaches for female FSD in IVF-ET.

## 2 Materials and methods

### 2.1 Study design

This was a cross-sectional observational study conducted from January 8, 2024, to April 26, 2024. The study setting was a tertiary hospital in Yunnan Province, China. Convenience sampling was used to select participants. This study has been approved by the hospital ethics committee (approval number: YYLW-2024–006). All methods were conducted in accordance with the Declaration of Helsinki.

### 2.2 Study population

Female patients undergoing IVF-ET assisted reproduction at a tertiary hospital in Yunnan Province, China, were selected as the study subjects. All participants were enrolled prior to oocyte retrieval. Inclusion criteria: ①Female patients diagnosed with infertility [9], aged ≥ 20 years, and undergoing IVF-ET assisted pregnancy; ②Have good cognitive and communication skills. Exclusion criteria: ①Application of donated eggs for IVF-ET assisted pregnancy; ③Previous chronic diseases related to FSD (FSD related chronic diseases refer to diseases that exist for a long time and affect individual sexual function, such as cardiovascular diseases, diabetes, malignant tumors, etc.); ④Undertaking sexual function therapy; ⑤Taking medication that affects sexual function; ⑥Suffering from abnormal genital development, cognitive impairment, or meeting the diagnostic criteria of the International Classification of Diseases-10, diagnosed with any psychological or mental illness; ⑦Individuals with intellectual or hearing impairments who are unable to understand the questionnaire content. Sample size calculation [10]: Following the recommended rule of 5–10 cases per variable for logistic regression analysis, and given that this study involves 25 variables, we calculated a required sample size of 125–250 cases. Considering 20% invalid questionnaires, the target sample size was 150–300 cases. Ultimately, 305 IVF-ET patients were included, exceeding the minimum requirement. Written informed consent was obtained from all participants before data collection. The informed consent form included the purpose of the study, confidentiality of personal information, and the right to withdraw at any time without any reason. The information provided was intended to help participants understand their rights and give them the opportunity to make an informed decision about participation in the study. All collected data were kept confidential, and the study did not involve minors.

### 2.3 Survey tools

#### 2.3.1 General information survey form.

The general information survey form was developed by researchers based on literature and expert opinions, including residence, educational level, age, duration of infertility, occupation, personal monthly income, number of embryo transfers, marital type, and number of pregnancies.

#### 2.3.2 Female sexual function index.

The Female Sexual Function Index (FSFI) questionnaire was developed by Rosen R et al [11], and the effectiveness of the Chinese version has been confirmed [12], including 19 questions and 6 dimensions, namely desire (items 1–2), arousal (items 3–6), lubrication (items 7–10), orgasm (items 11–13), satisfaction (items 11–14), and pain (items 17–19). The weight of questions 3–10 is 0.3, the weight of questions 1–2 is 0.6, and the weight of questions 11–19 is 0.4. The Likert 6-point scale was employed, with scores ranging from 0 to 5. The total score was calculated by multiplying the sum of scores across all dimensions by their respective weights, resulting in a range of 2–36 points. The lower the score, the more severe the sexual dysfunction. A total FSFI score of less than 25 indicates the presence of FSD. A libido score of less than 3.6 indicates low libido, a arousal score of less than 3.6 indicates difficulty in arousal, a lubrication score of less than 3.9 indicates difficulty in lubrication, an orgasmic score of less than 4.0 indicates difficulty in orgasm, a satisfaction score of less than 4.4 indicates decreased satisfaction, and a pain score of less than 4.4 indicates the presence of pain [13]. The Cronbach's α coefficient in this study was 0.971.

#### 2.3.3 The 10-item Kessler Psychological Distress Scale.

The 10-item Kessler Psychological Distress Scale (K10) was translated into Chinese by Zhou Chengchao et al [14]. The Chinese version has good reliability and validity, and can be popularized in Chinese people [15]. The scale consists of 10 items, ranging from “almost no” to “all times”, with a total score of 1–5 points. The higher the score, the more severe the psychological distress. The four score ranges of 10–15 points, 16–21 points, 22–29 points, and 30–50 points represent good, average, poor, and poor mental health, respectively [16]. In this study, the Cronbach's α coefficient was 0.940.

### 2.4 Data collection methods

After obtaining informed consent through explanation, the same researcher distributed scales to eligible patients. All scales were independently completed by patients and anonymously filled out and collected on the spot. At the end of the survey, the completeness and validity of the questionnaire were organized and checked. If the answers selected for each question in the questionnaire were the same or if the questionnaire was withdrawn for various reasons, they were discarded. A total of 321 questionnaires were distributed, with 305 valid responses and an effective response rate of 96%.

### 2.5 Statistical methods

Latent profile analysis (LPA) was performed using Mplus 8.3 with maximum likelihood estimation with robust standard errors (MLR). The model suitability indicators include: (1) Information indicators: Akaike Information Criterion (AIC), Bayesian Information Criterion (BIC), and Adjusted Bayesian Information Criterion (aBIC), with smaller values indicating better performance; (2) Classification indicator: Entropy ranges from 0 to 1, with a value greater than 0.8 indicating a classification accuracy of over 90%. (3) Likelihood ratio test: Lo-Mendell-Rubin (LMR) and Bootstrap like likelihood ratio test (BLRT), when the P-value is less than 0.05, indicate that a model with k categories is significantly better than a model with k-1 categories. The final number of latent classes was determined based on a combination of statistical fit indices, theoretical interpretability, and class size adequacy (each class comprising >5% of the sample). SPSS 27.0 was used to analyze the data. Quantitative data presented a normal distribution and were described using mean and standard deviation. Counting data were described using frequency and composition ratio; The associated factors of sexual function categories in female IVF-ET patients were analyzed using unordered multinomial logistic regression. Variance Inflation Factors (VIFs) were calculated to assess multicollinearity among predictors, with VIF values < 5 indicating no significant collinearity. The difference is statistically significant with P < 0.05.

## 3 Results

### 3.1 IVF-ET female sexual function index scale and The 10-item Kessler Psychological Distress Scale

The total score of the IVF-ET female sexual function index scale was (23.83 ± 7.95) points, and the average scores of six dimensions of desire, arousal, lubrication, orgasm, satisfaction, and pain are (3.08 ± 0.81), (3.79 ± 1.39), (4.46 ± 1.76), (4.25 ± 1.72), (4.56 ± 1.87), and (3.69 ± 1.39) points, respectively; The total score of the 10-item Kessler Psychological Distress Scale was (20.4 ± 7.97) points. There is a significant correlation between female sexual function and psychological distress (r = −0.198, P < 0.01).

### 3.2 Latent profile analysis results of female sexual function in IVF-ET

This study used the scores of six dimensions of the Women's Sexual Function Scale as the extrinsic indicators and conducted a latent profile analysis. Based on the number of categories, 1–5 latent profile models were established from small to large. As shown in Table 1, as the number of potential categories gradually increases, AIC, BIC, and aBIC gradually decrease, with Entropy values>0.800; When 4 or 5 potential categories are included, the AIC, BIC, and a BIC values are small, but the difference in LMR (P > 0.05) is not statistically significant. When the potential category is 3, the AIC, BIC, and aBIC values are small, and LMR (P < 0.05) and BLRT (P < 0.05) both show statistical significance. The grouping situation is reasonable, and the number of people in each group is greater than 5% of the total number of people. Therefore, three potential profiles are the optimal models. Specifically, the three-class solution was selected because: (1) AIC, BIC, and aBIC values showed substantial improvement from the 2-class to 3-class model with diminishing returns thereafter; (2) Entropy = 0.894 (>0.80) indicates excellent classification accuracy; (3) Both LMR (P = 0.0003) and BLRT (P < 0.001) were statistically significant for the 3-class vs. 2-class comparison, while LMR was not significant for the 4-class vs. 3-class comparison (P = 0.1411); and (4) All classes had proportions >5% of the total sample, ensuring adequate representation.

**Table 1:** Potential profile model fitting indicators for female sexual function in IVF-ET.

Profile	AIC	BIC	ABIC	LMR (P)	BLRT(P)	Entropy	probability
C1	5873.611	5918.254	5880.196	—	—	—	1
C2	3984.256	4054.942	3994.683	< 0.0001	< 0.000	1.000	21.0%/79.0%
C3	3679.750	3776.478	3694.019	0.0003	< 0.000	0.894	21.0%/40.9%/38.1%
C4	3595.495	3718.265	3613.605	0.1411	< 0.000	0.892	40.4%/21.0%/29.6%/9.0%
C5	3502.674	3651.487	3524.626	0.1159	< 0.000	0.914	3.0%/18.0%/39.9%/9.0%/30.2%

### 3.3 Naming of potential profiles of female sexual function in IVF-ET

**Fig 1:**
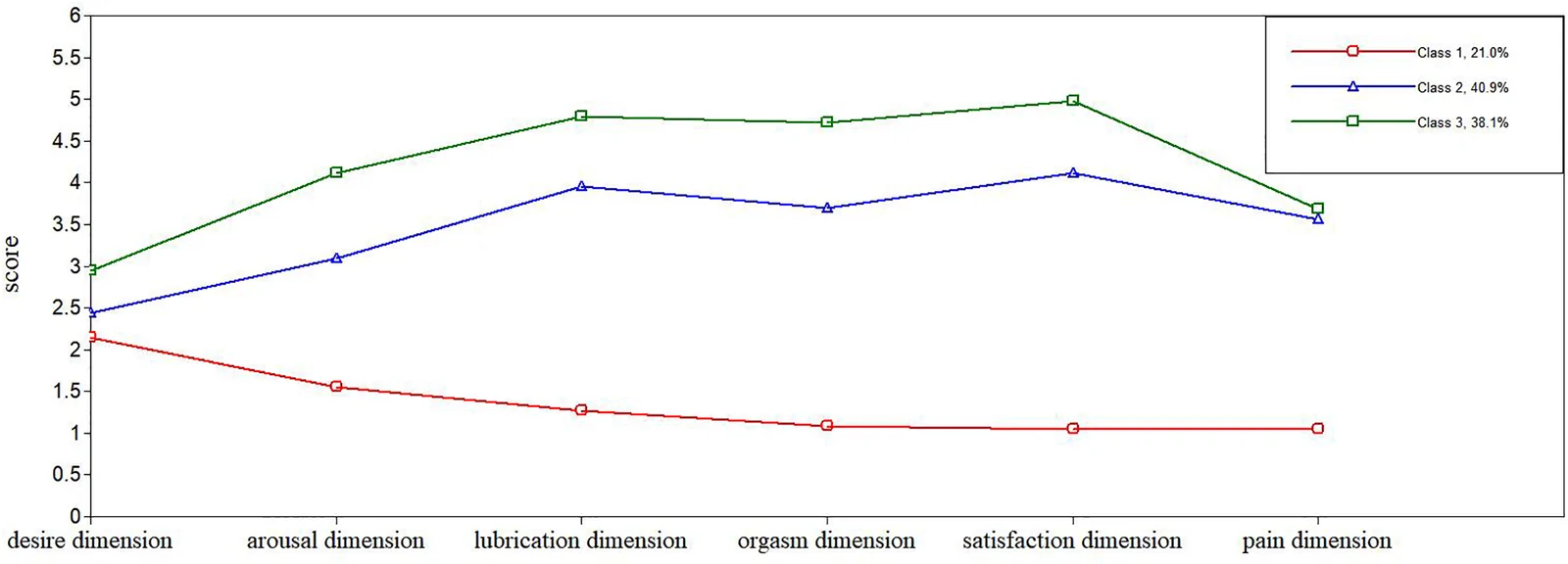
Distribution of three potential categories of female sexual function features in IVF-ET.

The probability of scores for the three profiles of female sexual function in IVF-ET on six dimensions is shown in Fig 1. (1) C1: 64 cases (21.0%), with the lowest scores in all six dimensions in this category. Therefore, Category 1 is named “low sexual function group”; (2) C2: There were 129 cases (40.9%) in this category, and the scores in 6 dimensions were at a moderate level. Therefore, category 2 was named “moderate sexual function group”; (3) C3: 112 cases (38.1%), with scores in 6 dimensions at a relatively high level, therefore category 3 is named “good sexual function group”.

### 3.4 Comparison of different dimensions of female sexual function potential categories in IVF-ET

There were statistically significant differences in the scores of the three potential categories in terms of desire, arousal, lubrication, orgasm, satisfaction, and pain (all P < 0.05). The group with low sexual function had the lowest score in the pain dimension (1.25 ± 0.27), while the group with moderate and good sexual function had the lowest scores in the desire dimension (2.92 ± 0.62) and (3.57 ± 0.7). The scores of the three potential categories in other dimensions are detailed in Table 2.

**Table 2:** Comparison of different dimensional scores for potential categories of female sexual function in IVF-ET.

group	Number of people	desire dimension	arousal dimension	lubrication dimension	orgasm dimension	satisfaction dimension	pain dimension
low sexual function group	64	2.58 ± 0.91	1.86 ± 1.05	1.52 ± 0.91	1.3 ± 0.34	1.25 ± 0.35	1.25 ± 0.27
Moderate sexual function group	129	2.92 ± 0.62	3.71 ± 0.76	4.76 ± 0.79	4.44 ± 0.72	4.95 ± 0.68	4.27 ± 0.59
Good sexual function group	112	3.57 ± 0.7	4.98 ± 0.61	5.79 ± 0.64	5.7 ± 0.5	6 ± 0.63	4.42 ± 0.67
F		45.038	327.495	646.435	1191.506	1297.196	737.139
P		< 0.001	< 0.001	< 0.001	< 0.001	< 0.001	< 0.001

### 3.5 Univariate analysis of general demographic data and three potential profiles of IVF-ET patients associated with sexual function

The results of univariate analysis showed that there were no statistically significant differences in occupation, personal monthly income, number of embryo transfers, and marital status among the three categories of IVF-ET patients (all P > 0.05); The comparison of sexual function scores among IVF-ET patients with different residence, ages, educational levels, duration of infertility, number of pregnancies, and psychological distress showed statistically significant differences (P < 0.05), as shown in Table 3.

**Table 3:** Single factor analysis of demographic data and potential profile associated factors of sexual function.

Variable	Number	low sexual function group	Moderate sexual function group	Good sexual function group	X^2^	P
Residence						
city	128	10 (15.6%)	44 (34.1%)	74 (66.1%)	48.225	< 0.001
countryside	177	54 (84.4%)	85 (65.9%)	38 (33.9%)		
Educational level						
Junior high school or below	106	53 (82.8%)	31 (24%)	22 (19.6%)	94.891	< 0.001
High school/vocational school	67	2 (3.1%)	34 (26.4%)	31 (27.7%)		
College/Undergraduate	125	5 (7.8%)	63 (48.8%)	57 (50.9%)		
Graduate student or above	7	4 (6.3%)	1 (0.8%)	2 (1.8%)		
Age (year)						
20–29	114	4 (6.3%)	37 (28.7%)	73 (65.2%)	117.559	< 0.001
> 29–39	168	40 (62.5%)	90 (69.8%)	38 (33.9%)		
> 39	23	20 (31.3%)	2 (1.6%)	1 (0.9%)		
duration of infertility (year)						
< 3	70	1 (1.6%)	22 (17.1%)	47 (42%)	69.322	< 0.001
3–5	107	14 (21.9%)	49 (38%)	44 (39.3%)		
> 5	128	49 (76.6%)	58 (45%)	21 (18.8%)		
occupation						
unemployed	87	17 (26.6%)	42 (32.6%)	28 (25%)	4.120	0.850
worker	51	11 (17.2%)	21 (16.3%)	19 (17%)		
farmer	45	13 (20.3%)	15 (11.6%)	17 (15.2%)		
employees	83	16 (25%)	35 (27.1%)	32 (28.6%)		
teacher	39	7 (10.9%)	16 (12.4%)	16 (14.3%)		
Personal monthly income (Yuan Renminbi/¥)						
< 3000	152	27 (42.2%)	73 (56.6%)	52 (46.4%)	7.791	0.252
3000-5999	119	28 (43.8%)	43 (33.3%)	48 (42.9%)		
> 5999-10000	28	9 (14.1%)	10 (7.8%)	9 (8%)		
> 10000	6	0 (0%)	3 (2.3%)	3 (2.7%)		
Number of embryo transfers						
0 times	228	45 (70.3%)	98 (76%)	85 (75.9%)	5.433	0.497
1 times	46	13 (20.3%)	16 (12.4%)	17 (15.2%)		
2 times	22	5 (7.8%)	12 (9.3%)	5 (4.5%)		
More than 3 times	9	1 (1.6%)	3 (2.3%)	5 (4.5%)		
Marital status						
first marriage	250	57 (89.1%)	107 (82.9%)	86 (76.8%)	4.298	0.118
remarriage	55	7 (10.9%)	22 (17.1%)	26 (23.2%)		
number of pregnancies						
0 times	218	15 (23.4%)	94 (72.9%)	109 (97.3%)	111.399	< 0.001
1 times	64	38 (59.4%)	25 (19.4%)	1 (0.9%)		
2 times	19	9 (14.1%)	9 (7%)	1 (0.9%)		
≥3 times	4	2 (3.1%)	1 (0.8%)	1 (0.9%)		

1 USD ≈ 7 Yuan Renminbi/¥.

### 3.6 Multivariate analysis of potential profiles of female sexual function in IVF-ET

Unordered multinomial logistic regression analysis was conducted using three categories of female sexual function in IVF-ET as the dependent variable (with the moderate sexual function group as the reference) and six statistically significant variables in univariate analysis (residence, age, education level, duration of infertility, number of pregnancies, and psychological distress) as independent variables. The results of the parallelism test showed that the X^2^ value was 22.915, P = 0.028. Therefore, unordered multinomial logistic regression analysis was used. The likelihood ratio test X^2^ = 318.068, P < 0.001, indicates a good fit of the model. VIF values for all predictors were below 2 (age: 1.187; duration of infertility: 1.179; number of pregnancies: 1.269; educational level: 1.199; residence: 1.104; psychological distress: 1.021), indicating no significant multicollinearity. The results of multivariate analysis showed that age, educational level, residence, duration of infertility, number of pregnancies, and psychological distress were the associated factors for different potential profiles of sexual function (all P < 0.05). Please refer to Tables 4 and 5 for details.

**Table 4:** Assignment of Independent Variables.

independent variable	Assignment method
Age (year)	20–29 = 1, > 29–39 = 2, > 39 = 3
Residence	City = 1, countryside = 2,
Educational level	Junior high school or below = 1, High school/vocational school = 2, College/Undergraduate = 3, Graduate student or above = 4
duration of infertility (year)	5 = 3
number of pregnancies	0 times = 1, 1 times = 2, 2 times = 3, ≥ 3 times = 4
psychological distress	Original value

**Table 5:** Unordered multinomial logistic regression analysis of potential categories of associated factors on female sexual function in IVF-ET.

Variable	low sexual function group vs. Moderate sexual function group (reference)			good sexual function group vs. Moderate sexual function group (reference)		
	P	OR	95%CI	P	OR	95%CI
Age (reference: > 39) (year)						
20-29	0.001	0.032	0.004–0.251	0.641	2.773	0.038–202.43
> 29-39	0.01	0.102	0.018–0.578	0.716	0.451	0.006–32.783
Residence (reference: countryside)						
City	0.393	0.616	0.203–1.871	< 0.001	5.058	2.447–10.457
Educational level (reference: Graduate student or above)						
Junior high school or below	0.436	0.355	0.026–4.812	0.746	0.616	0.033–11.488
High school/vocational school	0.005	0.013	0.001–0.274	0.957	0.923	0.05–16.997
College/Undergraduate	0.006	0.021	0.001–0.326	0.68	0.548	0.031–9.576
duration of infertility (reference: > 5) (year)						
< 3	0.016	0.033	0.002–0.532	0.001	4.963	1.917–12.845
3-5	0.299	0.565	0.192–1.659	0.244	1.596	0.726–3.504
number of pregnancies (reference: ≥ 3times)						
0 times	0.025	0.014	0–0.582	0.047	24.4	1.048–568.201
1 times	0.185	0.081	0.002–3.325	0.823	0.658	0.017–25.824
2 times	0.07	0.027	0.001–1.341	0.671	0.431	0.009–21.042
psychological distress	0.937	1.002	0.949–1.059	< 0.001	0.868	0.819–0.92

## 4 Discussion

### 4.1 Current status and characteristics of sexual function in female IVF-ET patients

The total score of sexual function in IVF-ET female patients was (23.83 ± 7.95) points, and the incidence of FSD was 37%. Among them, low sexual desire, difficulty in arousal, difficulty in vaginal lubrication, difficulty in orgasm, decreased sexual satisfaction, and pain during intercourse account for 57.4%, 33.4%, 25.9%, 28.2%, 25.9%, and 55.4%, respectively. Low sexual desire is the most common sexual dysfunction, similar to the research results of Lo et al [17], but there is a significant difference in the detection rate of FSD compared to the infertility population abroad [18]. Perhaps under the influence of traditional Chinese thought and culture, women who are more conservative in sexual issues are more ashamed to talk about; And most women lack understanding of their own sexual status, lack attention to sexual issues, and lack corresponding sexual knowledge. These conditions are more pronounced in infertile patients, leading to an underestimation of the actual occurrence of FSD in infertile patients in China.

The latent profile analysis results extracted three categories: low sexual function group (21.0%), moderate sexual function group (40.9%), and good sexual function group (38.1%). The total proportion of the group with low sexual function and the group with moderate sexual function is 61.9%, indicating that the sexual function level of female patients with IVF-ET is at a moderate or below level, which is consistent with the research results of Omani SamaniR et al [18]. It is necessary to focus on the sexual function level of female patients with IVF-ET and provide screening and support. IVF-ET women need to change their mindset, break free from the shackles of outdated thinking, value their sexual life quality, and promptly identify and actively treat sexual dysfunction, which is particularly important for maintaining marital relationships and IVF-ET treatment. Medical staff should incorporate patient sexual function into diagnosis, treatment, and nursing, strengthen patient sexual awareness education, promote sexual harmony, and actively provide scientific sexual health guidance for patients. Finally, enhance the cognitive and practical abilities of nurses in sexual health education, incorporate sexual health education into their training and education, and accurately evaluate and personalize patient sexual health through nursing practice. The group with good sexual function accounted for 38.1%, and the level of sexual function in this group was relatively high, with the highest score in the dimension of sexual satisfaction. This may be due to the positive coping strategies adopted by this group of patients, such as actively seeking professional information support, actively debugging, enhancing self-efficacy, and actively communicating with partners. Medical staff can help patients maintain a positive attitude, understand their actual needs and experiences, improve their quality of sexual life, and promote IVF-ET treatment. Therefore, cross-sectional analysis of sexual function in female IVF-ET patients can help clarify the characteristics of patients with different profiles, gain a deeper understanding of the differences in sexual function performance, and generate hypotheses for more accurate and personalized screening approaches in nursing practice.

### 4.2 Analysis of associated factors on potential categories of outcomes reported by IVF-ET patients

#### 4.2.1 Age and educational level.

This study shows that younger and more educated IVF-ET women are more likely to be included in the moderate sexual function group. The research results are consistent with those of scholars such as Daneshfar Z [19,20]. As age increases, ovarian and estrogen levels decrease, pelvic floor muscles relax [21,22], and sexual function changes to varying degrees, manifested as decreased libido, difficulty in vaginal lubrication, decreased sexual sensitivity, and painful intercourse [6]. And as they age, the fertility of infertile women decreases, their psychological burden becomes heavier, and they are more prone to FSD. A study found [23] that each additional year of age in infertile women was associated with a 26% increase in the risk of sexual dysfunction. But a foreign study suggests that young patients are under high stress and have a higher incidence of sexual dysfunction [24]. A higher level of education is a protective factor for FSD, which may be due to patients with higher levels of education being more open-minded, able to openly discuss sex with their spouses, express their feelings and demands, increase the frequency of communication, and have a better sexual experience; in addition, patients with higher education are able to actively seek rich FSD medical information, have a rapid ability to receive knowledge, and have a richer understanding of sexuality [25]. In this study, there was no statistically significant difference in educational level between the group with moderate sexual function and the group with good sexual function, indicating that reaching a certain educational level will not have further positive effects on female sexual function. Therefore, nursing staff should pay attention to elderly patients with lower educational levels, pay attention to comprehensive evaluation of their education level, establish sexual health education models [26], online consultation on social media [27] and other sexual health education methods, and improve the awareness of FSD among IVF-ET women; for IVF-ET women who have a heavy psychological burden, they should actively guide patients to dialectically view reproductive values, understand and accept themselves, and have a correct understanding of the laws of disease occurrence, development, and outcome.

#### 4.2.2 Residence.

This study shows that IVF-ET women residing in cities are more likely to belong to the group with good sexual function. Wang Junsong's [28] research found that only 15.7% of women use lubricants during sexual intercourse, with rural women significantly less likely to use lubricants than urban women. This may be due to the fact that women living in urban areas have rich access to knowledge, have a relatively better understanding of sex related knowledge, and are good at using knowledge to improve their sexual function status and enhance the quality of sexual life; Moreover, most rural women are greatly influenced by traditional beliefs, and sexual topics are often considered taboo. Usually, sexual behavior related issues are seen as private and shameful things. Some women are afraid of being labeled as “unfaithful” and dare not seek medical help, nor can they face and accept their sexual dysfunction problems. They even worry about being discriminated against if someone knows about their sexual dysfunction. Therefore, medical staff should strengthen the promotion and popularization of sexual knowledge for women in rural or remote areas, including normal anatomy, sexual physiological and psychological changes, sexual behavior patterns, etc., so that they can face sexual difficulties correctly, establish correct sexual concepts, actively seek medical treatment, and improve the quality of sexual life.

#### 4.2.3 duration of infertility and number of pregnancies.

This study shows that the shorter the duration of infertility and the fewer pregnancies, the less likely it is to be included in the low sexual function group. Infertile women who receive assisted reproduction need routine medication, as medication can easily lead to FSD in infertile women [29], which may be related to long-term medication causing endocrine disorders and decreased sexual function; During IVF-ET treatment, repeated laboratory tests, strict periodic life plans, and other factors can also disrupt sexual harmony between couples. Therefore, the shorter the infertility period, the less affected the medication and treatment on sexual activity. In addition, patients with longer duration of infertility have higher expectations for fertility. Sexual activity aimed at conception results in a lack of pleasure for both partners, decreased sexual satisfaction, and a tendency to generate negative emotions [30], which can increase the occurrence of FSD. Pregnancy and childbirth can reduce sexual function through physical and mental health and disease factors [31–33], such as hormonal imbalances, neuromodulatory disorders, negative emotions, pelvic floor dysfunction, vaginal injuries or infections. Therefore, nursing staff should focus on IVF-ET patients who have a long treatment time and a large number of births, adopt multi-dimensional, high-quality care, and early identify and improve the patient's adverse sexual life status; For patients with pelvic floor muscle relaxation or dysfunction, provide sexual health education, guide pelvic floor muscle exercise, and encourage early medical treatment.

#### 4.2.4 psychological distress.

After being diagnosed with infertility, IVF-ET patients are prone to significant psychological discomfort, which is equivalent to the pressure caused by diseases such as cancer [34], seriously affecting their physical and mental health and sexual life. Therefore, this study analyzed the correlation between psychological distress and sexual function in IVF-ET women. The results showed that the total score of the 10-item Kessler Psychological Distress Scale was (20.4 ± 7.97) points, indicating that the psychological health status of IVF-ET patients was average; Moreover, psychological distress is a factor associated with the potential category of sexual function in IVF-ET women, meaning that IVF-ET women with psychological distress are more likely to belong to the moderate sexual function group. Perhaps IVF-ET patients are prone to anxiety, depression, and other emotions [29], and patients with strong reproductive desires no longer view sexual activity as an intimate way between couples, but instead view conception as the only result of sexual activity. Some patients even only engage in it during ovulation, resulting in a decrease in female satisfaction with IVF-ET, increased sexual pressure, and limited sexual function [35,36], manifested as difficulties in sexual arousal, discomfort during intercourse, and obstacles to orgasm. Therefore, nursing staff should respect and emphasize the value of women, guide patients to change their reproductive concepts, and reduce sexual pressure. For patients with difficulty in sexual arousal or sexual hyperactivity disorder, encourage both spouses to communicate and encourage more, build confidence, and gradually achieve sexual pleasure; For patients who experience discomfort during sexual intercourse, appropriate lubricants can be used to alleviate it.

The hormonal treatments inherent to IVF-ET protocols, including gonadotropins, GnRH agonists, and GnRH antagonists, may directly influence sexual function through their effects on the hypothalamic-pituitary-ovarian axis. These medications can induce rapid fluctuations in estradiol and progesterone levels, which are known to affect vaginal lubrication, sexual desire, and arousal [37]. Furthermore, the intensive nature of IVF-ET treatment—involving frequent clinic visits, transvaginal ultrasound monitoring, and blood draws—can exacerbate psychological stress and fatigue, further compromising sexual function [38]. The bidirectional relationship between infertility and sexual dysfunction warrants attention: infertility-related stress can precipitate or worsen FSD, while FSD may complicate fertility treatment by creating marital tension and reducing the frequency of sexual activity [7]. These complex interrelationships underscore the need for integrated psychosocial and medical care for IVF-ET patients.

Notwithstanding these contributions, several limitations of this study should be acknowledged to contextualize the findings appropriately. First, the cross‑sectional design precludes causal inferences; the identified associations between sociodemographic/clinical factors and sexual function profiles do not establish temporality or causation. Second, the wide confidence intervals observed for some regression estimates—particularly for subgroups defined by educational level and number of pregnancies—indicate limited precision due to sparse data in certain categories, and these findings should therefore be interpreted with caution. Third, participants were enrolled prior to oocyte retrieval, and sexual function may differ at other stages of IVF‑ET treatment (e.g., embryo transfer, luteal phase, or post‑pregnancy confirmation). Fourth, while the sample size was adequate for the primary analyses, it was insufficient for detailed subgroup analyses. In light of these limitations, future multi‑center, longitudinal studies with larger and more diverse samples are warranted to validate these findings and to evaluate the clinical utility of profile‑based screening approaches in routine fertility care.

## 5 Conclusions

In this sample of Chinese women undergoing IVF-ET, three distinct profiles of sexual function were identified: low sexual function group (21.0%), moderate sexual function group (40.9%), and good sexual function group (38.1%). Age, educational level, residence, duration of infertility, number of pregnancies, and psychological distress were significantly associated with the likelihood of belonging to different sexual function profiles. Reproductive centers should be aware of the heterogeneity in sexual function among IVF-ET patients. Medical staff may use these findings to identify at-risk populations and develop hypothesis-driven screening strategies.
